# Supplementary figures and images for: Natural killer (NK) cell-derived extracellular-vesicle shuttled microRNAs control T cell responses
Source: eLife. 2022 Jul 29;11:e76319. doi: 10.7554/eLife.76319 (PMC9366747; doi:10.7554/eLife.76319)

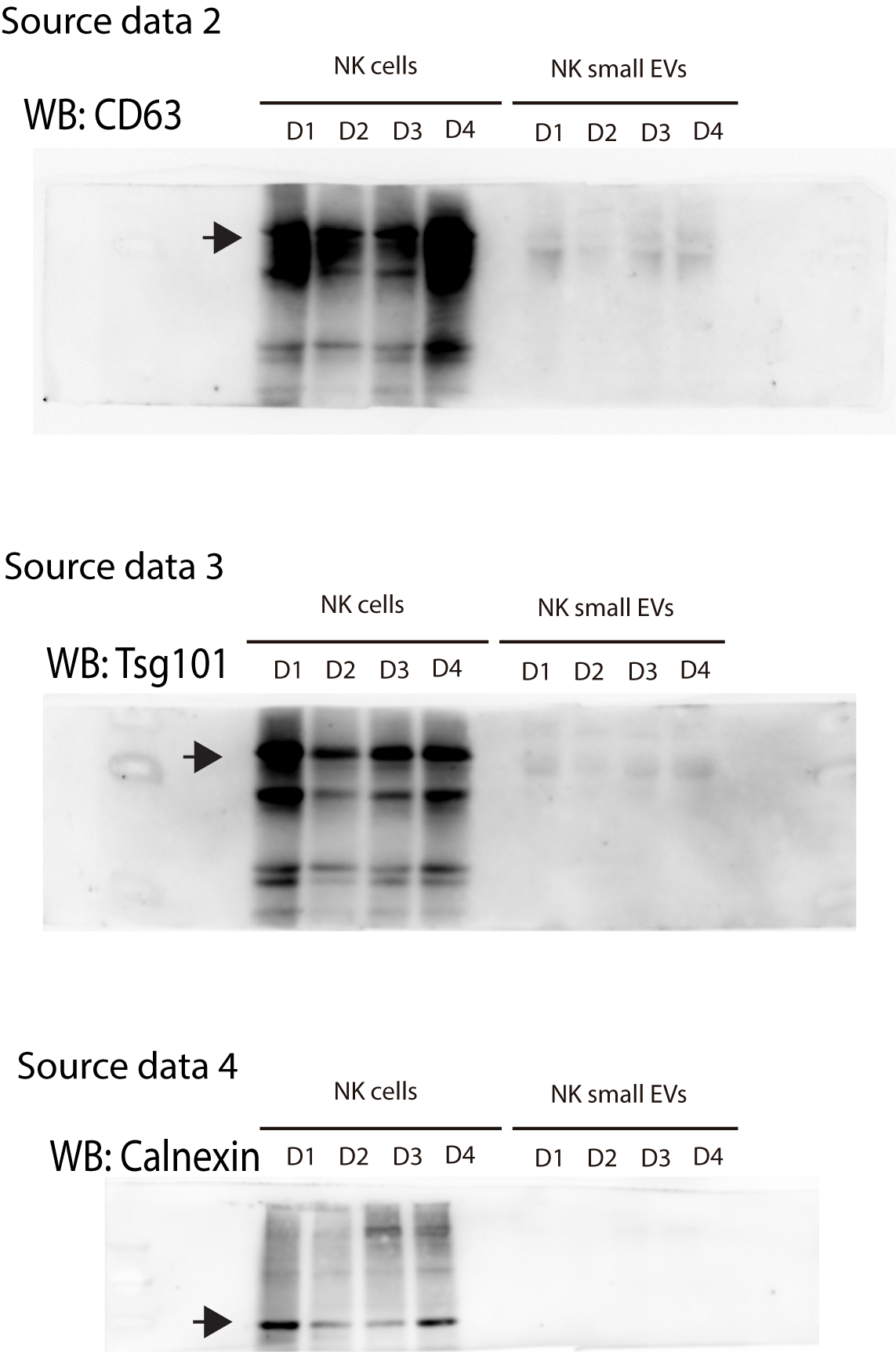

Supplement: Figure 1—figure supplement 1—source data 1. [file elife-76319-fig1-figsupp1-data1.tif]

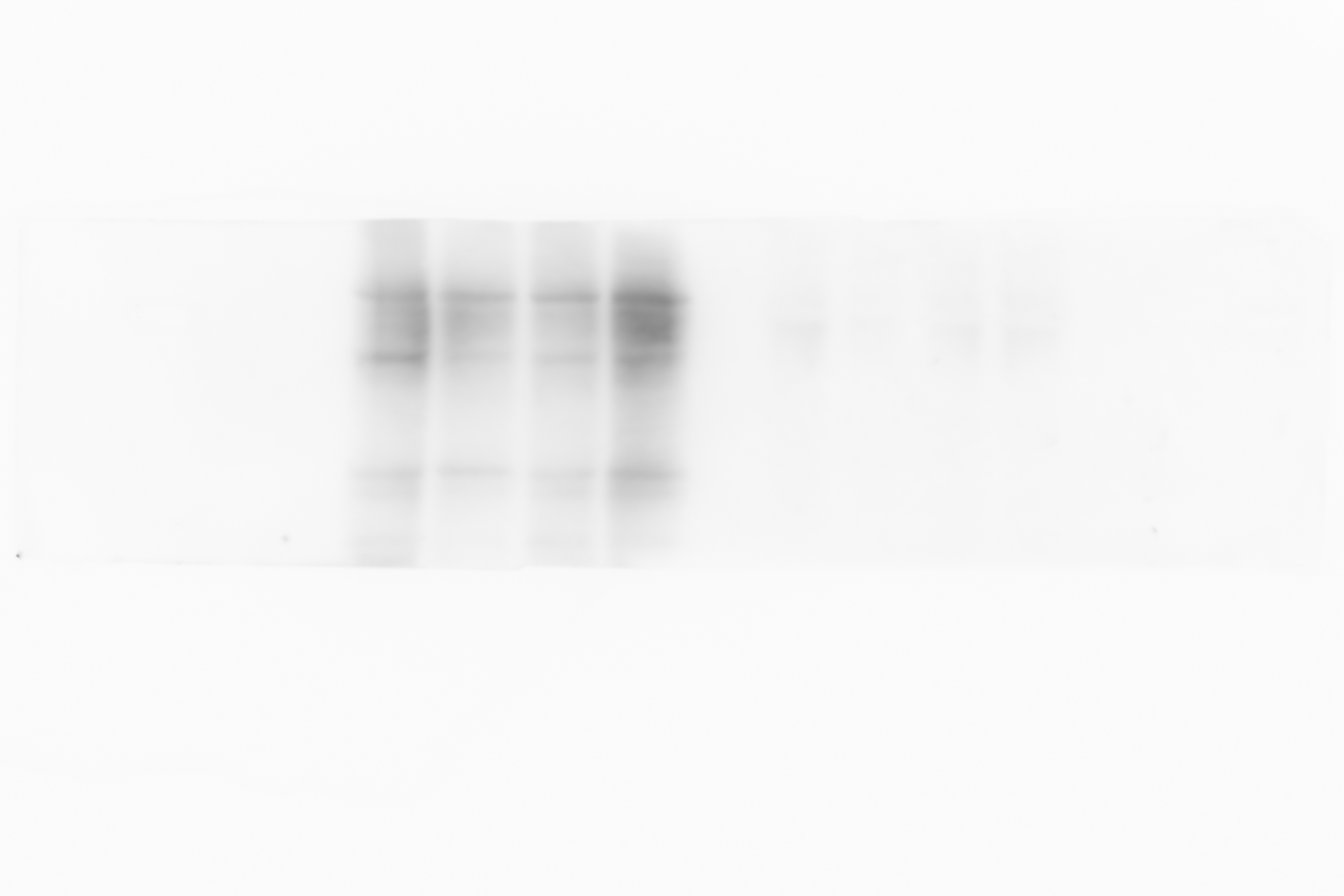

Supplement: Figure 1—figure supplement 1—source data 2. [file elife-76319-fig1-figsupp1-data2.tif]

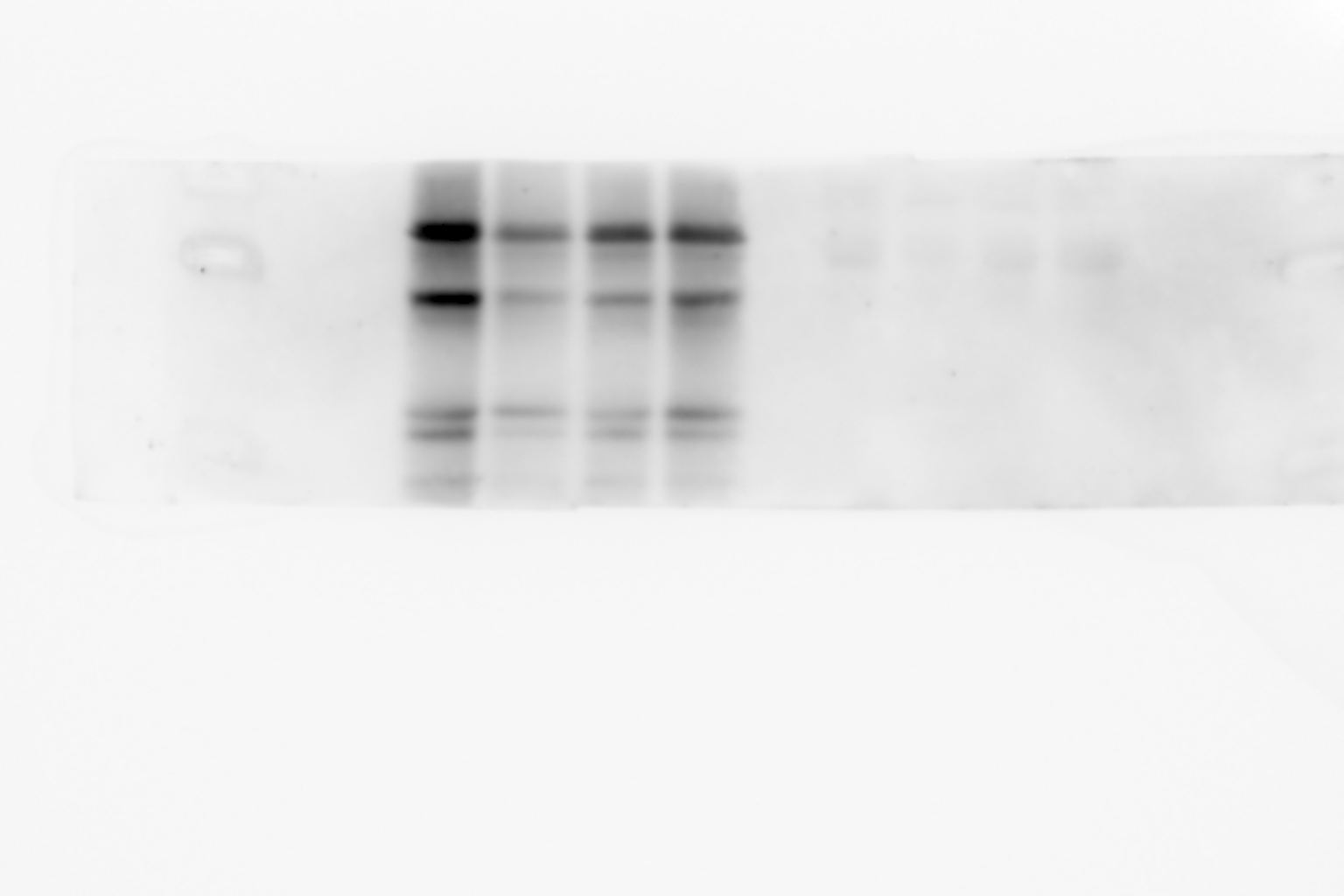

Supplement: Figure 1—figure supplement 1—source data 3. [file elife-76319-fig1-figsupp1-data3.tif]

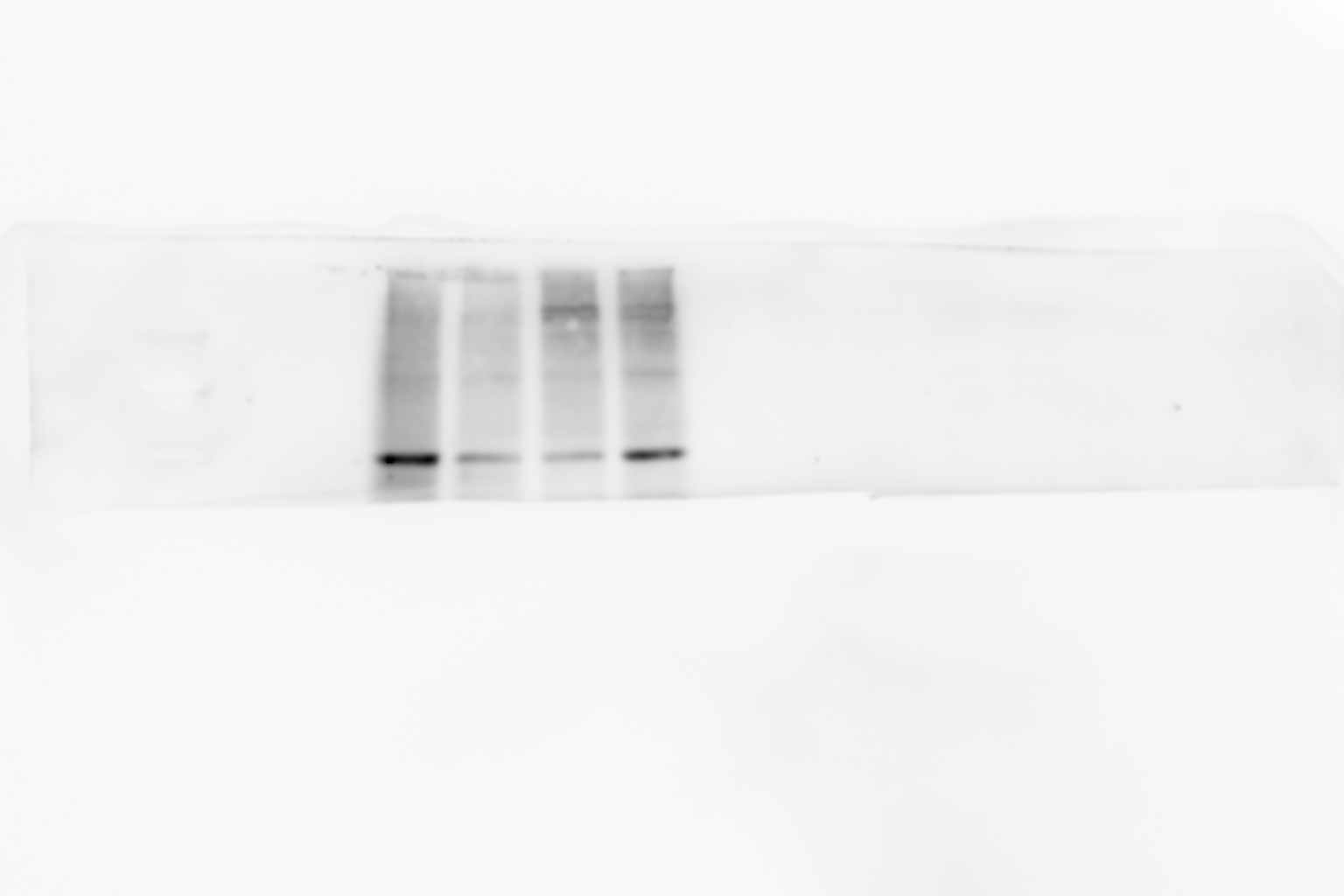

Supplement: Figure 1—figure supplement 1—source data 4. [file elife-76319-fig1-figsupp1-data4.tif]

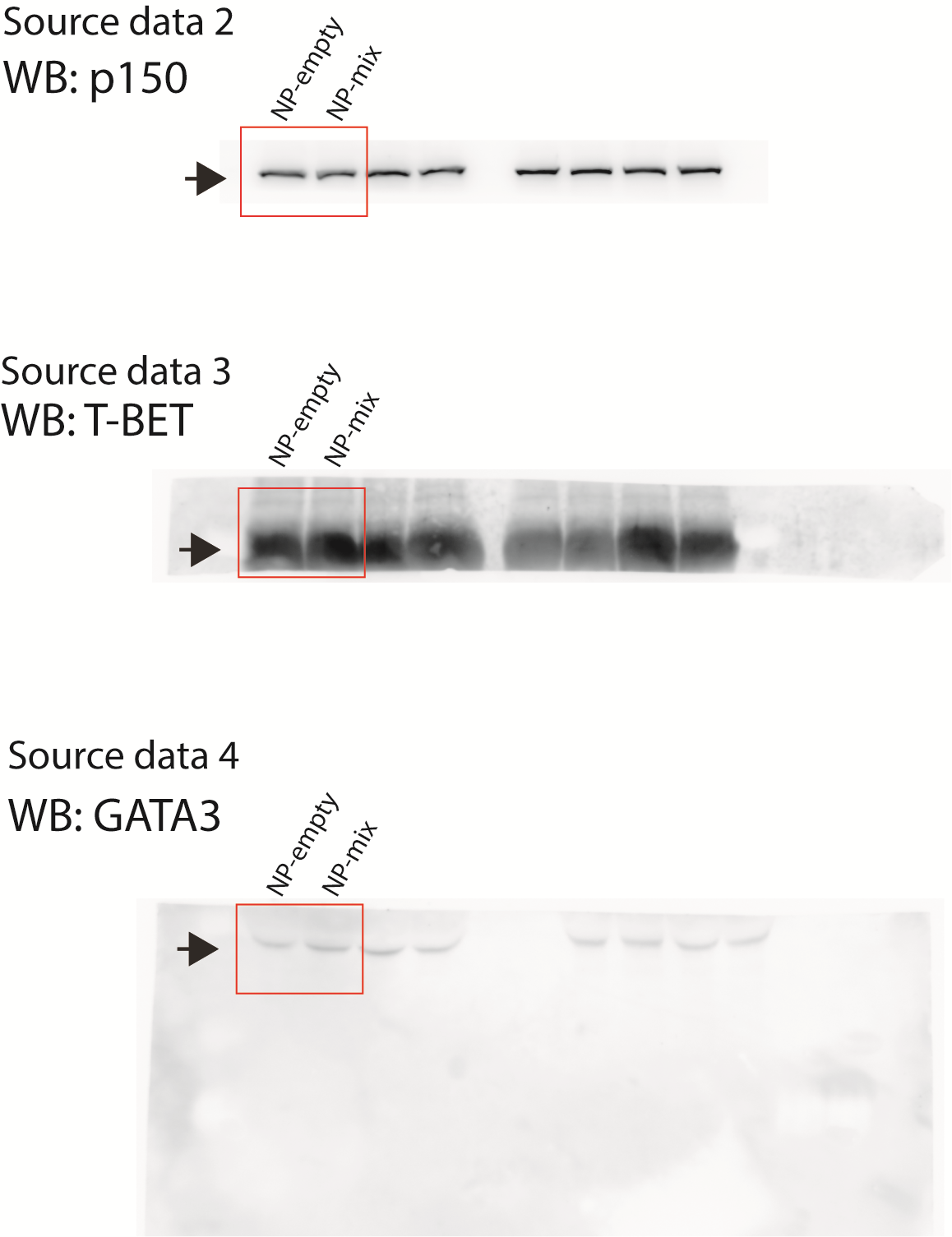

Supplement: Figure 3—figure supplement 1—source data 1. [file elife-76319-fig3-figsupp1-data1.tif]

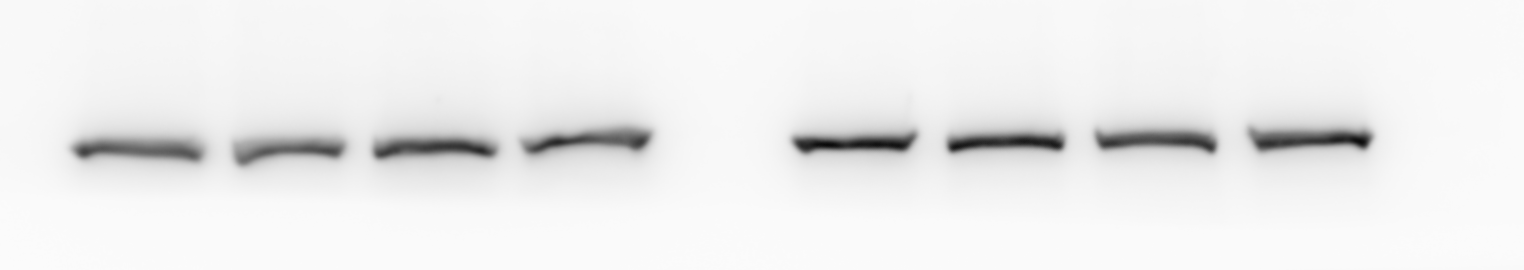

Supplement: Figure 3—figure supplement 1—source data 2. [file elife-76319-fig3-figsupp1-data2.tif]

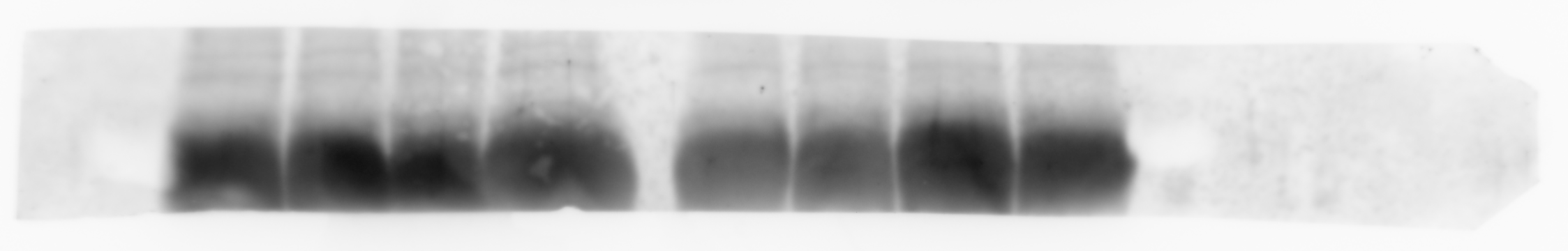

Supplement: Figure 3—figure supplement 1—source data 3. [file elife-76319-fig3-figsupp1-data3.tif]

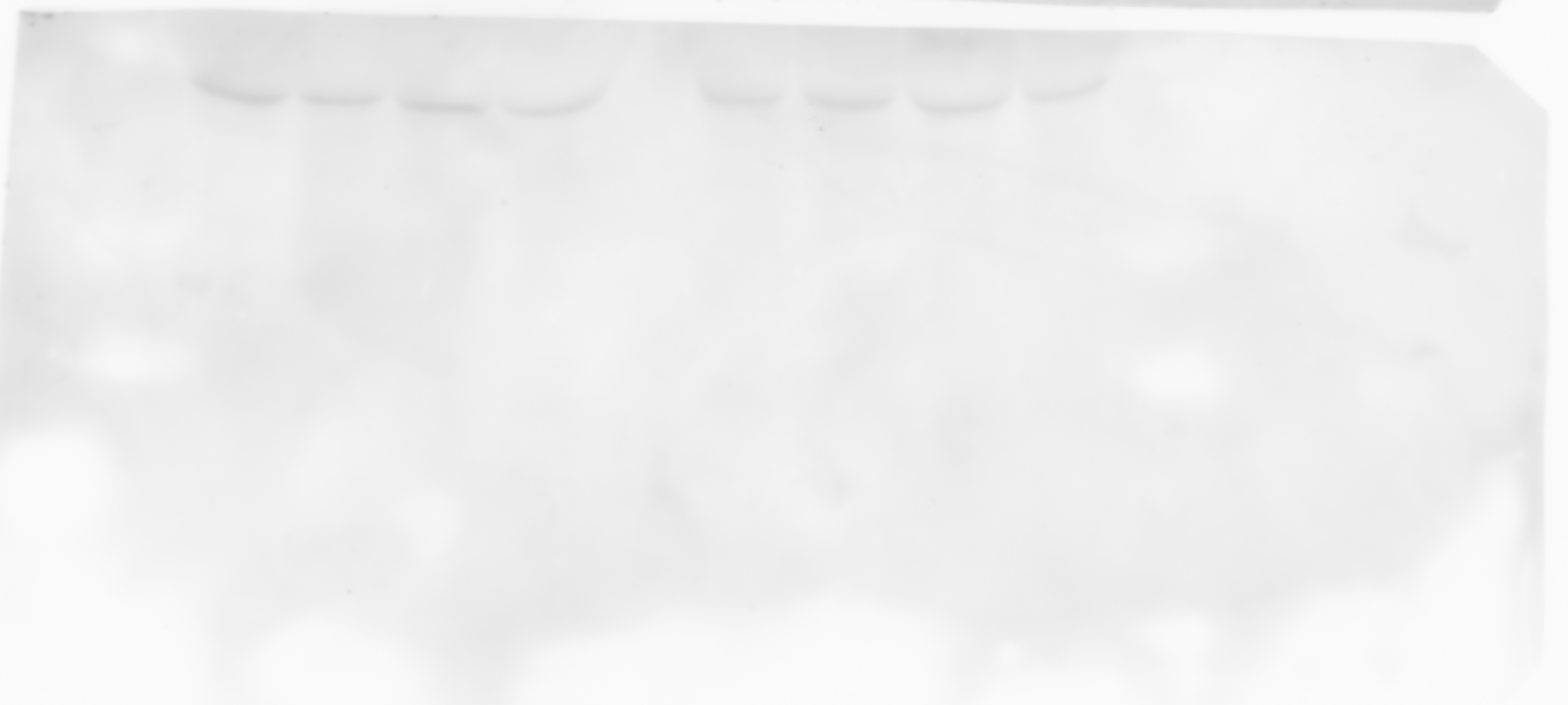

Supplement: Figure 3—figure supplement 1—source data 4. [file elife-76319-fig3-figsupp1-data4.tif]

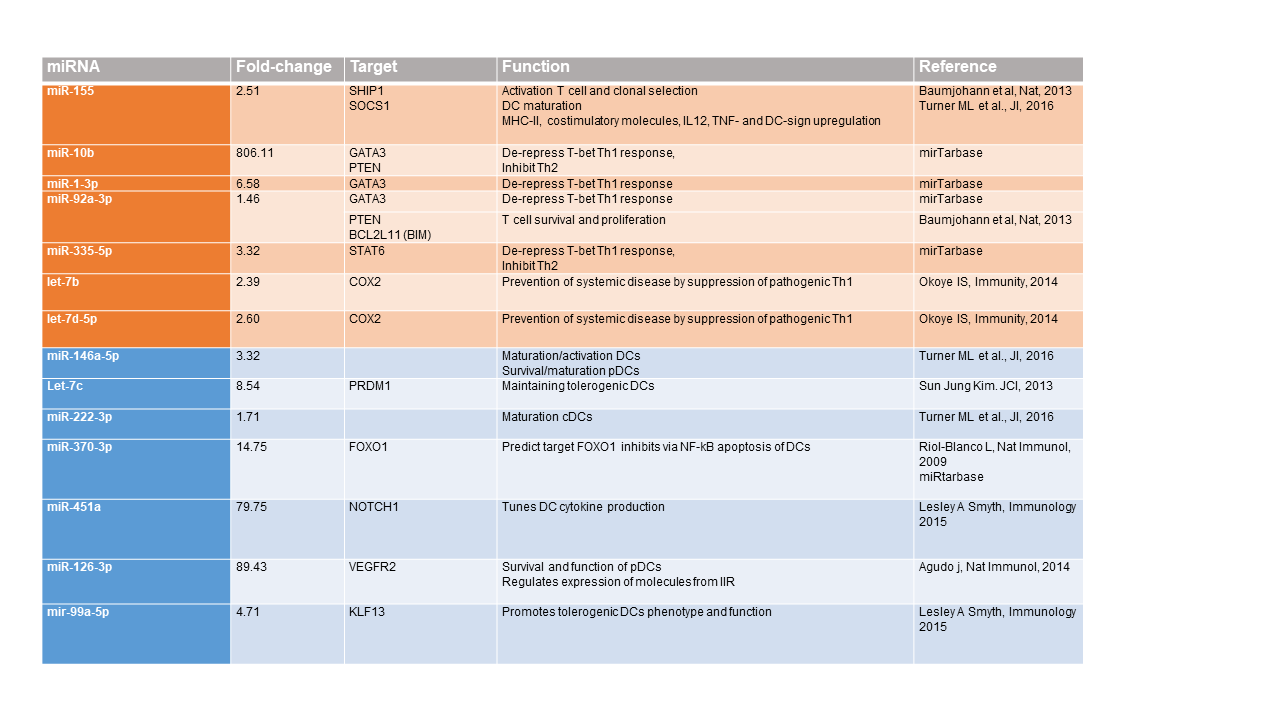

Supplement: Supplementary file 2. — Table summarizes putative mRNA targets for selected NK-EV miRNAs identified by in silico analyses, using the miRTarBase database. T cell function-related miRNAs are highlighted in orange and dendritic cell (DC)-related miRNAs in blue. [file elife-76319-supp2.tif]
