# Supplementary material for: Natural killer (NK) cell-derived extracellular-vesicle shuttled microRNAs control T cell responses
Source: eLife. 2022 Jul 29;11:e76319. doi: 10.7554/eLife.76319 (PMC9366747; doi:10.7554/eLife.76319)
Supplement: Supplementary file 4. [file elife-76319-supp4.pptx]

## Slide 1
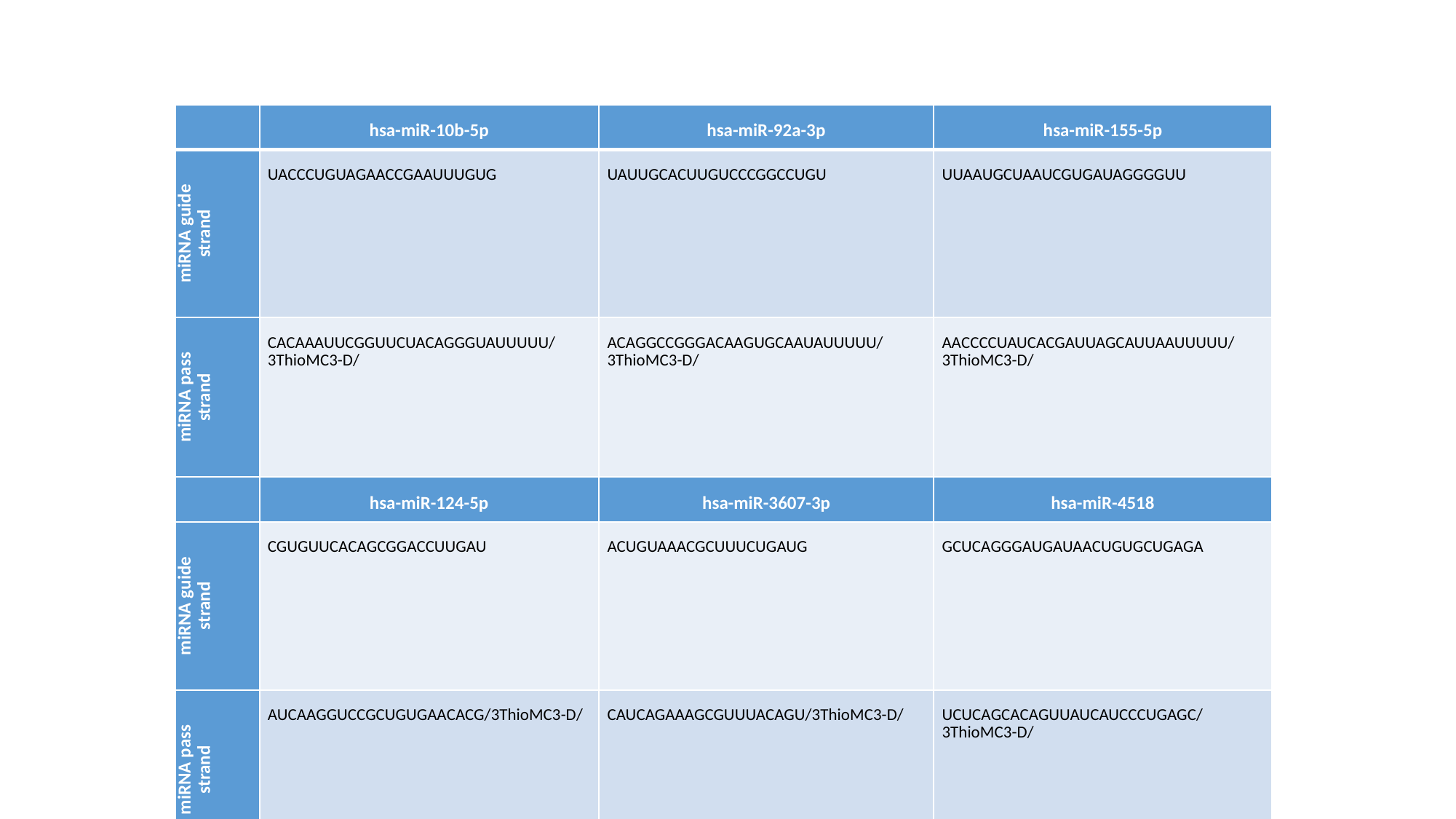

| | hsa-miR-10b-5p | hsa-miR-92a-3p | hsa-miR-155-5p |
| --- | --- | --- | --- |
| miRNA guide strand | UACCCUGUAGAACCGAAUUUGUG | UAUUGCACUUGUCCCGGCCUGU | UUAAUGCUAAUCGUGAUAGGGGUU |
| miRNA pass strand | CACAAAUUCGGUUCUACAGGGUAUUUUU/3ThioMC3-D/ | ACAGGCCGGGACAAGUGCAAUAUUUUU/3ThioMC3-D/ | AACCCCUAUCACGAUUAGCAUUAAUUUUU/3ThioMC3-D/ |
| | hsa-miR-124-5p | hsa-miR-3607-3p | hsa-miR-4518 |
| miRNA guide strand | CGUGUUCACAGCGGACCUUGAU | ACUGUAAACGCUUUCUGAUG | GCUCAGGGAUGAUAACUGUGCUGAGA |
| miRNA pass strand | AUCAAGGUCCGCUGUGAACACG/3ThioMC3-D/ | CAUCAGAAAGCGUUUACAGU/3ThioMC3-D/ | UCUCAGCACAGUUAUCAUCCCUGAGC/3ThioMC3-D/ |
